# Associations between five anthropometric indices and fecal incontinence: A cross-sectional study based on the 2005 to 2010 NHANES data

**DOI:** 10.1097/MD.0000000000049139

**Published:** 2026-06-05

**Authors:** Zhijun Ye, Guorong Huang, Xiaoyun Lan, Lijuan Huang, Erdong Chen, Yanghua Hong, Xue Zhang, Limin Tao

**Affiliations:** a Department of Colorectal Surgery, Zhangzhou Traditional Chinese Medicine Hospital, Zhangzhou, China; b Department of Gastroenterology, Zhangzhou Traditional Chinese Medicine Hospital, Zhangzhou, China; c Department of Critical Care, Zhangzhou Traditional Chinese Medicine Hospital, Zhangzhou, China.

**Keywords:** A Body Shape Index, conicity index, fecal incontinence, National Health and Nutrition Examination Survey, relative fat mass, waist-to-height ratio

## Abstract

Fecal incontinence (FI) is a common condition that impairs quality of life. While the association between general adiposity, measured by body mass index (BMI), and FI is inconsistent, central adiposity may be more relevant. Indices such as the waist-to-height ratio (WHtR) and novel anthropometric indices, including the Conicity Index (CI), A Body Shape Index (ABSI), and relative fat mass (RFM), aim to better quantify central fat distribution. However, their association with FI is not well characterized. We aimed to comprehensively evaluate and compare the associations of these 5 indices with FI. This cross-sectional study analyzed data from the 2005 to 2010 cycles of the National Health and Nutrition Examination Survey, including 10,097 participants. FI was defined based on the Bowel Health Questionnaire as any involuntary loss of mucus, liquid, or solid stool within the previous month. Multivariate logistic regression was used to evaluate the association between the 5 anthropometric indices and FI. We used restricted cubic splines for dose–response, conducted sensitivity and subgroup analyses, and compared association strengths using the area under the receiver operating characteristic curve. The prevalence of FI was 8.4%. After full adjustment for confounders (Model 3), each standard deviation increase in the *z*-score of CI (odds ratio = 1.28, 95% CI: 1.18–1.39), ABSI (1.28, 1.18–1.39), RFM (1.29, 1.13–1.46), and WHtR (1.15, 1.06–1.24) was positively associated with FI, but not BMI (1.05, 0.98–1.13). The dose–response relationships were linear (*P*-value for nonlinearity: CI = .08, ABSI = .63, RFM = .31, WHtR = .25). Subgroup analyses indicated interactions by age, BMI, and chronic diarrhea, but positive associations persisted in most subgroups. CI and ABSI demonstrated modest discriminatory ability for FI (area under the receiver operating characteristic curve = 0.620 and 0.613, respectively), exceeding WHtR (0.595), RFM (0.585), and BMI (0.548). CI, ABSI, RFM, and WHtR, but not BMI, were positively associated with FI, with CI and ABSI showing relatively stronger associations. These findings suggest that central fat distribution is more strongly related to FI than overall obesity. Given their modest predictive accuracy, future studies should validate these findings and assess their clinical utility.

## 1. Introduction

Fecal incontinence (FI) is defined as the involuntary passage of solid or liquid stool.^[1]^ As a common pelvic floor dysfunction, FI affects 7%–15% of community-dwelling adults and seriously affects quality of life.^[2]^ Previous studies have shown that age is a risk factor for FI, with an increased prevalence of 75% (odds ratio [OR]: 1.75, 95% confidence interval [CI]: 1.39–2.20) in adults older than 60 years compared with those younger than 60 years.^[3]^ The prevalence in females (9.1%) was higher than that in males (7.4%).^[3]^ In addition, dementia (hazard ratio: 1.7, 95% CI: 1.6–1.8),^[4]^ anal sphincter injury (relative risk = 2.44, 95% CI: 1.92–3.09),^[5]^ and diarrhea (OR: 2.94, 95% CI: 1.16–7.45)^[6]^ were also risk factors for FI. FI is often accompanied by psychological distress, social withdrawal, and depression, which increase the burden of care and the social and economic burden and bring challenges to clinical and public health.^[7–11]^ Identification of modifiable risk factors for FI is important for prevention and early intervention.

The relationship between anthropometric indices and FI has attracted increasing attention. The association between overall obesity and FI remains unclear, while body mass index (BMI), the most common measure, has shown inconsistent associations in different studies,^[12–14]^ whereas measures of central fat distribution have shown more consistent associations. For example, waist-to-height ratio (WHtR), a simple measure of central obesity, was associated with a 75% (OR = 1.75; 95% CI: 1.09–2.79) increased risk of FI per unit increase in a recent cross-sectional study.^[15]^ In addition to WHtR, novel anthropometric indices, including the conicity index (CI), A Body Shape Index (ABSI), and relative fat mass (RFM), have been developed to more precisely quantify body morphology and fat distribution.^[16–18]^ CI and ABSI quantify body size and central obesity using specific formulas that combine waist circumference (WC), height, and weight.^[18–20]^ RFM estimates total body fat percentage based on the ratio of height to WC, making it sensitive to central fat accumulation.^[21]^ These indices also predict cardiometabolic risk and mortality better than BMI.^[22–24]^ Previous studies have shown that these indices may be correlated with other pelvic floor dysfunctions, such as urinary incontinence and overactive bladder.^[25,26]^ However, there is a lack of studies correlating these indices with FI, and a direct comparison with WHtR has not been performed. Mechanistically, visceral adipose tissue may impair pelvic floor and intestinal function by increasing intra-abdominal pressure and promoting systemic inflammation, suggesting a potential biological link between these indices and FI. Therefore, it is necessary to systematically evaluate and compare these indices to provide richer insights into the relationship between obesity and FI, which can be used to identify modifiable FI risk factors and develop preventive and early intervention strategies.

This study used data from the National Health and Nutrition Examination Survey (NHANES, 2005–2010) to assess and compare the associations between 5 anthropometric indices (CI, ABSI, RFM, WHtR, and BMI) and FI. To evaluate the relationship between these indices and FI, we used multivariate regression, restricted cubic spline (RCS), subgroup, and receiver operating characteristic (ROC) curve analyses. The results aim to provide new insights into the association between anthropometric indices and FI and to inform risk assessment strategies.

## 2. Materials and methods

### 2.1. Data source and ethical considerations

The data for this study were derived from publicly available data from the 2005 to 2006, 2007 to 2008, and 2009 to 2010 cycles of the NHANES conducted by the National Center for Health Statistics.^[27]^ NHANES is a repeated cross-sectional survey conducted in continuous 2-year cycles since 1999. It is designed to assess the health and nutritional status of a nationally representative sample of the US non-sheltered civilian population, covering all 50 states and the District of Columbia. Its sampling design is complex, and a stratified multistage probability sampling method is adopted to ensure that the sample can effectively represent the national population. We selected these 3 survey cycles because, during these years, NHANES administered the Bowel Health Questionnaire (BHQ), which contains the information necessary to assess FI status. The NHANES study protocol was approved by the National Center for Health Statistics Institutional Review Board, and written informed consent was obtained from all participants before data collection. No additional institutional review board approval was required because this was a secondary analysis of fully de-identified publicly available data. The NHANES dataset is rich and provides details on 5 core components: demographic profiles, physical examination records, laboratory test results, health questionnaire responses, and dietary assessment data. Such high-quality data are an important cornerstone of public health research and policy development both in the United States and globally. All the data used in the study were obtained free of charge from the NHANES official website (https://www.cdc.gov/nchs/nhanes/index.html). The reporting of this study follows the STROBE guidelines.

### 2.2. Study protocol and participants

**Figure 1.**
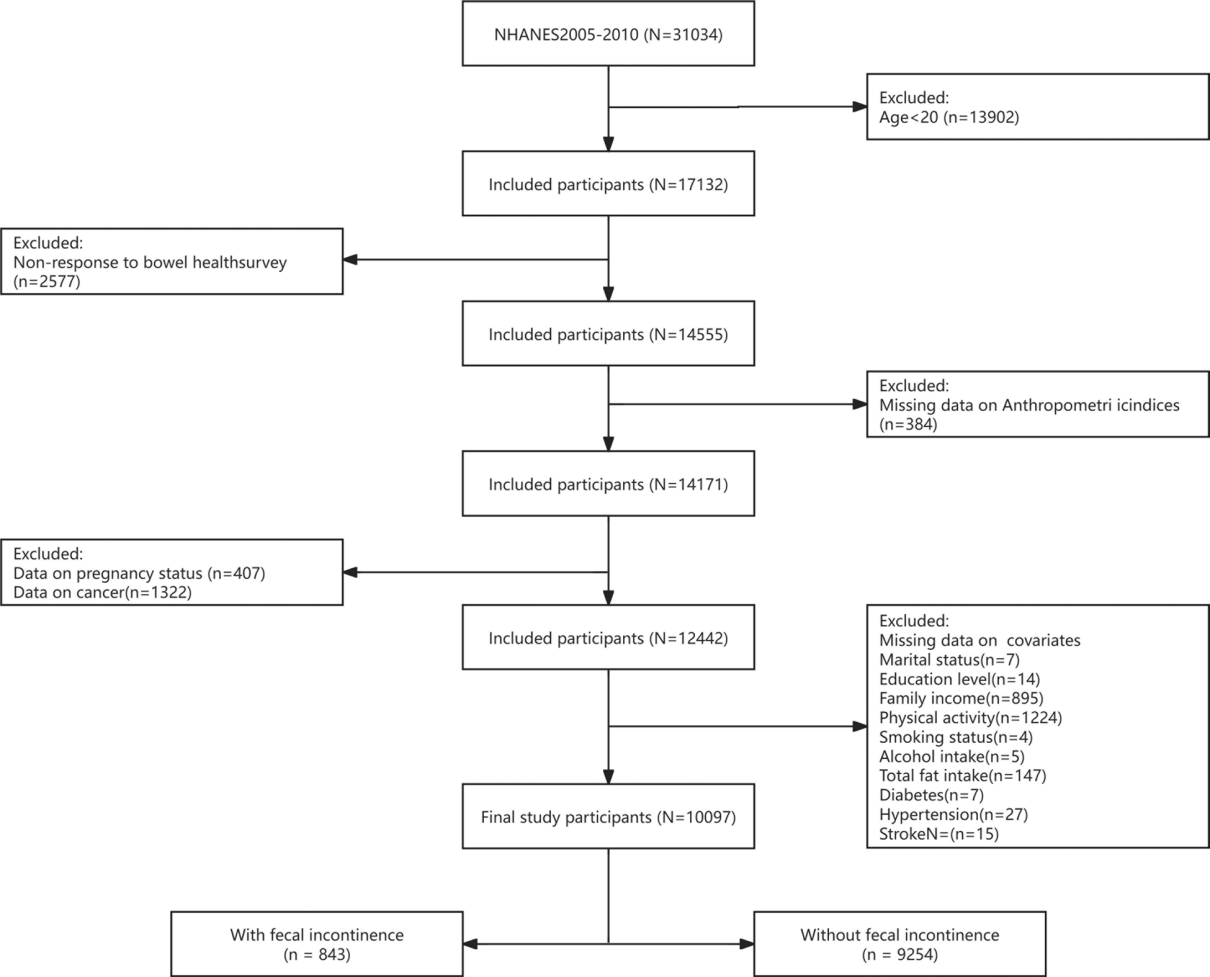
Participant selection process (National Health and Nutrition Examination Survey, 2005 to 2010 cycles). NHANES = National Health and Nutrition Examination Survey.

This study included NHANES participants aged 20 years or older who had complete anthropometric and BHQ data. The exclusion criteria included incomplete data on anthropometric indices (height, weight, and WC), FI, current pregnancy, history of cancer, or insufficient details on covariates. Figure 1 presents an overview of the participant screening process.

### 2.3. Anthropometric data collection and derivation

Extensive health data, including self-reported information and standardized physical measurements, were compiled from the NHANES database. At mobile screening centers, trained health technicians adhered to standardized procedures to measure participants’ weight, height, and WC, with each measurement being conducted collaboratively by a technician and recorder. The following anthropometric indices were derived from the measurements: CI, ABSI, RFM, WHtR, and BMI. The equations are as follows^[25,28]^:


$$CI=WC\;\left(m\right)/0.109/{\left[WT\;\left(kg\right)/height\;\left(m\right)\right]}^{0.5}$$

$$ABSI=WC\;\left(m\right)/\left[BMI^{2/3}\left(kg/m^{2}\right)\;\times \;height^{1/2}\;\left(m\right)\right]$$

For men:RFM=64−{20×[Height(m)/wc(m)]}

For women:RFM=76−{20×[Height(m)/wc(m)]}

WHtR=WC(m)/height(m)

$$BMI=WT\;\left(kg\right)/height^{2}\left(m^{2}\right)$$

### 2.4. Assessment of FI

FI was assessed using data from the BHQ in the NHANES cycles. The BHQ includes items adapted from the Rockwood Fecal Incontinence Severity Index to assess the frequency of involuntary stool leakage.^[29,30]^ Participants were asked, “Over the past 30 days, how often have you had any loose or liquid stools?” and “Over the past 30 days, how often have you had any solid stools?” Response options for each item were: never, 1–3 times per month, 1 time per week, ≥2 times per week, 1 time per day, or ≥2 times per day. In this study, FI was operationally defined as any episode of involuntary expulsion of mucoid, liquid, or solid feces within the past 30 days, explicitly excluding cases involving only gas incontinence.^[15,31]^

### 2.5. Assessment of covariates

The covariates included in this study were selected based on previous studies and their clinical relevance.^[9,15,32]^ Demographic characteristics included sex (male/female), age, and race (non-Hispanic White, non-Hispanic Black, Mexican American, or other race). Socioeconomic factors included education level (<9 years, 9–12 years, and >12 years), marital status (married or living with a partner and living alone), and family poverty-to-income ratio (PIR) categorized into 3 groups: <1.3 (low income), 1.3–3.5 (moderate income), and >3.5 (high income). Lifestyle factors included the following: smoking status was categorized as never smokers (had smoked < 100 cigarettes in their lifetime), former smokers (had smoked ≥ 100 cigarettes but were not current smokers), and current smokers; alcohol intake (yes/no, determined by annual intake of ≥12 standard drinks); total fat intake (based on 24-h dietary recall data); and physical activity level. Physical activity levels were classified into 4 categories: sedentary (no regular physical activity), insufficiently active (regular activity below the guideline minimum, <500 metabolic equivalent of task [MET] min/week), moderately active (500–1000 MET-min/week), and highly active (>1000 MET-min/week). Health-related covariates included the following: comorbidity status and depression. Comorbid conditions (diabetes, hypertension, stroke, and cancer) were treated as dichotomous variables (yes/no), with information confirmed through the question, “Has a doctor ever told you that you have [condition]?” Depression was assessed using the 9-item Patient Health Questionnaire (Cronbach α = 0.89, indicating excellent internal consistency), with total scores calculated by summing the item responses and defined as depressed at a 9-item Patient Health Questionnaire ≥ 10. Additionally, bowel health-related factors, including diarrhea (Bristol Stool Form Scale Type 6 or 7) and constipation (Bristol Stool Form Scale Type 1 or 2), were incorporated.

### 2.6. Statistical analysis

All analyses were conducted on the final sample of 10,097 participants. Participants with missing data for any exposure, outcome, or covariate were excluded. Characteristics of included and excluded participants were compared. NHANES sampling weights were not applied in the analyses. Descriptive statistics were used to summarize the demographic and clinical characteristics of the total sample and to compare participants with and without FI. Categorical variables were presented as frequencies and percentages (%), and associations were evaluated using Pearson chi-square tests for comparisons between groups. Continuous variables were initially examined for distributional characteristics using the Shapiro–Wilk normality test. Normally distributed data were reported as mean ± standard deviation (SD), whereas non-normally distributed data were presented as median and interquartile range. Differences in baseline characteristics between participants with and without FI were analyzed based on data distribution. Independent *t* tests were applied to normally distributed continuous variables, and the Mann–Whitney *U* test was used for non-normally distributed data. Statistical significance was set at *P* < .05. Missing data from a large sample were handled through listwise deletion. All anthropometric indices were standardized through *z*-score conversion using the following formula: *z*-score = (index − index_mean)/index_sd (Table S1, Supplemental Digital Content).

Independent associations were assessed while sequentially controlling for potential confounders. We created 3 multivariate logistic regression models to explore the association between anthropometric indices (CI, ABSI, RFM, WHtR, and BMI) and FI. The covariates for adjustment were selected based on prior literature and clinical relevance, and sequential models were used to progressively adjust for demographic, lifestyle, and then clinical factors to assess the stability of the associations. Model 1 (crude model) estimated the unadjusted association. Model 2 (partially adjusted model) was adjusted for age, sex, race, education, marital status, PIR, physical activity level, smoking status, alcohol intake, and total fat intake to account for demographic and socioeconomic confounders. Model 3 (fully adjusted) was also adjusted for diabetes, hypertension, depression, chronic diarrhea, constipation, and stroke to examine the associations after accounting for these clinical conditions. Sensitivity analyses were performed by categorizing the anthropometric indices into quartiles to verify the statistical robustness of the results.

A 4-knot (knots at the 5th, 35th, 65th, and 95th percentiles) RCS model was used to assess potential dose–response relationships, with smoothed curve fitting applied to determine the linearity of these associations.

Subgroup analyses were performed to assess whether the association between the anthropometric indices and FI differed across population strata. Heterogeneity was assessed using interactions in the regression model. Subgroup variables included age (<65 years vs ≥65 years), sex (male vs female), smoking status (never/current vs former), alcohol consumption status (nondrinker vs drinker), BMI (<25/25–30 vs >30 kg/m^2^), and the presence of hypertension, diabetes, depression, stroke, or chronic diarrhea.

The discriminatory performance of each anthropometric index for FI was evaluated using ROC curves and area under the curve (AUC) comparisons. All analyses were performed using R 4.2.2 (http://www.R-project.org; The R Foundation) and Free Statistics software (version 2.1; Beijing Free Clinical Medical Technology Co., Ltd., Beijing, China). Two-tailed tests were used, with a *P*-value of <.05 defined as a statistically significant difference.

## 3. Results

### 3.1. Participant selection

This study commenced with 31,034 participants from the NHANES 2005 to 2010 cycles. As detailed in Figure 1 and Table S2, Supplemental Digital Content, participants were sequentially excluded due to: age <20 years (n = 13,902); incomplete bowel health survey (n = 2577); missing anthropometric data (n = 384); current pregnancy (n = 407); history of cancer (n = 1322); and missing data on major covariates, including marital status (n = 7), education level (n = 14), PIR (n = 895), physical activity (n = 1224), smoking status (n = 4), alcohol intake (n = 5), total fat intake (n = 147), diabetes (n = 7), hypertension (n = 27), and stroke (n = 15). In total, 10,097 participants were included in the final analysis.

The excluded participants were older than the included participants (33.18% vs 19.15% aged ≥65 years), and a higher proportion were women (54.05% vs 49.15%). Crucially, the excluded group had significantly higher mean values for measures of central adiposity. These included CI (1.32 vs 1.30), ABSI (0.08 vs 0.08), RFM (36.47 vs 34.97), and WHtR (0.60 vs 0.59) (all *P* < .001). The excluded group had higher levels of exposure factors and a greater burden of FI-related risk factors. This suggests that the final sample may not be fully representative of the original NHANES population.

### 3.2. Baseline characteristics

Table 1 shows the baseline characteristics of the 10,097 participants, grouped according to their FI status. FI was observed in 8.4% of the patients (n = 843). The FI cohort demonstrated a significantly higher average age (proportion aged ≥65 years: 32.38% vs 17.95%) and a significantly greater proportion of females (54.92% vs 48.63%) than the participants without FI. Significant differences were also observed in race distribution, with a higher proportion of non-Hispanic whites in the FI group (55.16% vs 48.27%, respectively). No significant differences were found in the educational level (*P* = 0.12) or constipation prevalence (*P* = 0.95). However, the FI group had a higher proportion of individuals living alone (44.01% vs 38.48%), with a low family income (32.38% vs 28.82%), sedentary physical activity (27.88% vs 20.14%), and current smoking status (30.13% vs 23.23%). The prevalence of diabetes, hypertension, depression, chronic diarrhea, and stroke was significantly higher in the FI group (all *P* < .001). The mean CI, ABSI, RFM, WHtR, and BMI were significantly higher in the FI group than in the non-FI group (all *P* < .001).

**Table 1:** Characteristics of the study participants by fecal incontinence status (National Health and Nutrition Examination Survey, 2005–2010 cycles).

Characteristics	Total (n = 10,097)	Without FI (n = 9254)	With FI (n = 843)	*P-value*
Age (yr), n (%)				<.001
<65	8163 (80.85)	7593 (82.05)	570 (67.62)	
≥65	1934 (19.15)	1661 (17.95)	273 (32.38)	
Sex, n (%)				<.001
Male	5134 (50.85)	4754 (51.37)	380 (45.08)	
Female	4963 (49.15)	4500 (48.63)	463 (54.92)	
Race, n (%)				<.001
Non-Hispanic White	4932 (48.85)	4467 (48.27)	465 (55.16)	
Non-Hispanic Black	2044 (20.24)	1882 (20.34)	162 (19.22)	
Mexican American	1800 (17.83)	1682 (18.18)	118 (14)	
Others	1321 (13.08)	1223 (13.22)	98 (11.63)	
Education level (yr), n (%)				.12
<9	1024 (10.14)	933 (10.08)	91 (10.79)	
9–12	3948 (39.10)	3595 (38.85)	353 (41.87)	
>12	5125 (50.76)	4726 (51.07)	399 (47.33)	
Marital status, n (%)				.002
Married or living with a partner	6165 (61.06)	5693 (61.52)	472 (55.99)	
Living alone	3932 (38.94)	3561 (38.48)	371 (44.01)	
Poverty income ratio, n (%)				<.001
Low	2940 (29.12)	2667 (28.82)	273 (32.38)	
Medium	3844 (38.07)	3498 (37.8)	346 (41.04)	
High	3313 (32.81)	3089 (33.38)	224 (26.57)	
Physical activity, n (%)				<.001
Sedentary	2099 (20.79)	1864 (20.14)	235 (27.88)	
Insufficient	1692 (16.76)	1525 (16.48)	167 (19.81)	
Moderate	1227 (12.15)	1148 (12.41)	79 (9.37)	
High	5079 (50.30)	4717 (50.97)	362 (42.94)	
Smoking status, n (%)				<.001
Never	5418 (53.66)	5026 (54.31)	392 (46.5)	
Current	2404 (23.81)	2150 (23.23)	254 (30.13)	
Former	2275 (22.53)	2078 (22.46)	197 (23.37)	
Alcohol intake, n (%)	7417 (73.46)	6816 (73.65)	601 (71.29)	.14
Total fat intake (g/d), median (IQR)	71.46 (47.01, 102.78)	71.57 (46.92, 102.71)	70.62 (48.50, 104.25)	.72
Chronic diseases				
Diabetes, n (%)	1039 (10.29)	883 (9.54)	156 (18.51)	<.001
Hypertension, n (%)	2676 (26.50)	2329 (25.17)	347 (41.16)	<.001
Depression, n (%)	848 (8.40)	681 (7.36)	167 (19.81)	<.001
Chronic diarrhea, n (%)	745 (7.38)	595 (6.43)	150 (17.79)	<.001
Constipation, n (%)	737 (7.30)	675 (7.29)	62 (7.35)	.95
Stroke, n (%)	281 (2.78)	229 (2.47)	52 (6.17)	<.001
Anthropometric indices				
Weight·kg, mean (SD)	82.18 ± 21.01	82.06 ± 20.93	83.51 ± 21.91	.06
Height, cm, mean (SD)	168.19 ± 10.15	168.32 ± 10.12	166.77 ± 10.31	<.001
WC, cm, mean (SD)	98.64 ± 16.10	98.30 ± 16.04	102.45 ± 16.20	<.001
CI, mean (SD)	1.30 ± 0.09	1.30 ± 0.09	1.33 ± 0.09	<.001
ABSI, mean (SD)	0.08 ± 0.01	0.08 ± 0.01	0.08 ± 0.01	<.001
RFM, mean (SD)	34.97 ± 8.57	34.76 ± 8.55	37.28 ± 8.47	<.001
WHtR, mean (SD)	0.59 ± 0.10	0.59 ± 0.10	0.62 ± 0.10	<.001
BMI, kg/m^2^, mean (SD)	28.97 ± 6.60	28.88 ± 6.57	29.92 ± 6.86	<.001

Data are presented as number (percentage) for categorical variables and as mean ± standard deviation (SD) or median (interquartile range, IQR) for continuous variables.

ABSI = A Body Shape Index, BMI = body mass index, CI = conicity index, FI = fecal incontinence, RFM = relative fat mass, WC = waist circumference, WHtR = waist-to-height ratio.

### 3.3. Association of anthropometric indices with FI

The associations between the *z*-scores of the anthropometric indices and FI are summarized in Table 2. When analyzed as continuous variables, all 5 indices showed positive associations with FI in the unadjusted model (Model 1, all *P* < .001). In the fully adjusted model (Model 3), significant positive associations persisted for CI (OR per 1-SD increase: 1.28, 95% CI: 1.18–1.39), ABSI (OR: 1.28, 95% CI: 1.18–1.39), RFM (OR: 1.29, 95% CI: 1.13–1.46), and WHtR (OR: 1.15, 95% CI: 1.06–1.24) (all *P* < .001). In contrast, the association for BMI was no longer statistically significant in Model 3 (OR: 1.05, 95% CI: 0.98–1.13; *P* = .18).

**Table 2:** Multivariable logistic regression analyses of the association between anthropometric indices and fecal incontinence (National Health and Nutrition Examination Survey, 2005–2010 cycles).

Characteristic	Model 1*		Model 2†		Model 3‡	
	OR (95% CI)	*P-value*	OR (95% CI)	*P-value*	OR (95% CI)	*P-value*
Continuous						
CI *z*-score	1.52 (1.42–1.64)	<.001	1.41 (1.31–1.53)	<.001	1.28 (1.18–1.39)	<.001
ABSI *z*-score	1.49 (1.39–1.6)	<.001	1.37 (1.27–1.49)	<.001	1.28 (1.18–1.39)	<.001
RFM *z*-score	1.35 (1.25–1.45)	<.001	1.52 (1.35–1.71)	<.001	1.29 (1.13–1.46)	<.001
WHtR *z*-score	1.35 (1.26–1.44)	<.001	1.28 (1.19–1.37)	<.001	1.15 (1.06–1.24)	<.001
BMI *z*-score	1.16 (1.08–1.24)	<.001	1.15 (1.08–1.23)	<.001	1.05 (0.98–1.13)	.19
Quartiles						
CI *z*-score						
Q1 [−3.542 to −0.685]	Reference		Reference		Reference	
Q2 [−0.685 to 0.044]	1.33 (1.05–1.7)	.02	1.34 (1.05–1.71)	.02	1.26 (0.98–1.61)	.07
Q3 [0.004–0.686]	2.05 (1.64–2.57)	<.001	1.94 (1.54–2.45)	<.001	1.7 (1.34–2.16)	<.001
Q4 [0.686–6.591]	2.89 (2.33–3.58)	<.001	2.43 (1.93–3.07)	<.001	1.86 (1.46–2.37)	<.001
*P* for trend		<.001		<.001		<.001
ABSI *z*-score						
Q1 [−4.843 to −0.661]	Reference		Reference		Reference	
Q2 [−0.661 to −0.004]	1.38 (1.09–1.75)	.01	1.38 (1.09–1.76)	.01	1.25 (0.98–1.59)	.08
Q3 [−0.003–0.655]	1.84 (1.46–2.3)	<.001	1.79 (1.42–2.26)	<.001	1.58 (1.25–2)	<.001
Q4 [0.655–7.298]	2.8 (2.26–3.47)	<.001	2.32 (1.83–2.93)	<.001	1.9 (1.5–2.42)	<.001
*P* for trend		<.001		<.001		<.001
RFM *z*-score						
Q1 [−3.151 to −0.731]	Reference		Reference		Reference	
Q2 [−0.731 to −0.075]	1.2 (0.96–1.5)	.11	1.21 (0.96–1.52)	.11	1.07 (0.84–1.35)	.58
Q3 [−0.074 to 0.816]	1.43 (1.15–1.77)	.001	1.72 (1.32–2.25)	<.001	1.38 (1.05–1.82)	.02
Q4 [0.816–2.546]	2.14 (1.75–2.63)	<.001	2.78 (2.02–3.82)	<.001	1.88 (1.35–2.62)	<.001
*P* for trend		<.001		<.001		<.001
WHtR *z*-score						
Q1 [−2.409 to −0.718]	Reference		Reference		Reference	
Q2 [−0.718 to −0.083]	1.23 (0.98–1.55)	.08	1.2 (0.95–1.52)	.12	1.15 (0.9–1.46)	.26
Q3 [−0.083 to 0.617]	1.65 (1.32–2.05)	<.001	1.52 (1.21–1.91)	<.001	1.35 (1.07–1.7)	.01
Q4 [0.617–4.734]	2.44 (1.98–3)	<.001	2.08 (1.68–2.59)	<.001	1.6 (1.27–2.01)	<.001
*P* for trend		<.001		<.001		<.001
BMI *z*-score						
Q1 [−2.391 to −0.702]	Reference		Reference		Reference	
Q2 [−0.701 to −0.151]	1.03 (0.83–1.28)	.78	1.04 (0.84–1.29)	.74	1.01 (0.81–1.25)	.96
Q3 [−0.149 to 0.502]	1.22 (0.99–1.5)	.06	1.21 (0.98–1.49)	.08	1.08 (0.87–1.34)	.47
Q4 [0.503–7.133]	1.52 (1.24–1.85)	<.001	1.51 (1.23–1.85)	<.001	1.2 (0.97–1.49)	.09
*P* for trend		<.001		<.001		.07

95% CI = 95% confidence interval, ABSI = A Body Shape Index, BMI = body mass index, CI = conicity index, OR = odds ratio, PIR = poverty-to-income ratio, RFM = relative fat mass, WHtR = waist-to-height ratio.

* Model 1: No covariates were adjusted.

† Model 2: adjusted for age, sex, race, education level, marital status, PIR, physical activity, smoking status, alcohol intake, and total fat intake.

‡ Model 3: adjusted for age, sex, race, education level, marital status, PIR, physical activity, smoking status, alcohol intake, total fat intake, diabetes, hypertension, depression, chronic diarrhea, constipation, and stroke.

Sensitivity analyses using quartile categorization of the indices are presented in Table 2. In the fully adjusted model (Model 3), participants in the highest quartile (Q4) of CI, ABSI, RFM, and WHtR had significantly higher odds of FI compared to those in the lowest quartile (Q1), with ORs of 1.86 (95% CI: 1.46–2.37), 1.90 (95% CI: 1.50–2.42), 1.88 (95% CI: 1.35–2.62), and 1.60 (95% CI: 1.27–2.01), respectively (all *P* < .001). For BMI, the OR comparing Q4 to Q1 was 1.52 (95% CI: 1.24–1.85; *P* < .001) in Model 1, and 1.20 (95% CI: 0.97–1.49; *P* = 0.092) in Model 3. The *P* for trends across BMI quartiles was <.001 in Model 1 and .07 in Model 3.

**Figure 2.**
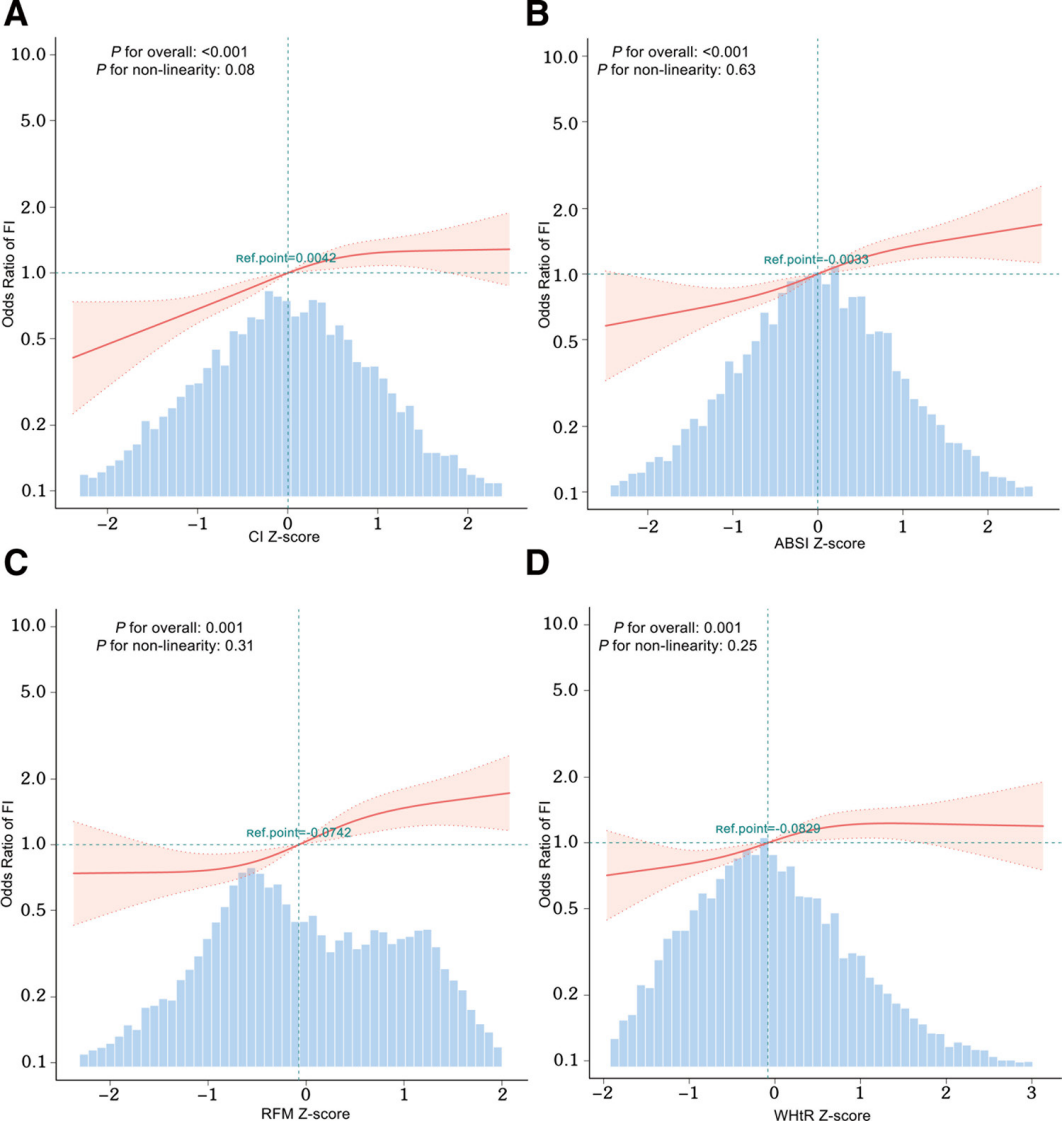
Dose–response relationships between anthropometric indices and fecal incontinence (National Health and Nutrition Examination Survey, 2005–2010 cycles). The models were adjusted for age, sex, race, education level, marital status, PIR, physical activity, smoking status, alcohol intake, total fat intake, diabetes, hypertension, depression, chronic diarrhea, constipation, and stroke. To reduce the effect of extreme values, spline models and plots were truncated to the 0.5 to 99.5th percentile range (lower and upper 0.5% excluded). The solid red line shows the fitted adjusted odds ratio (OR), and the light red shaded band indicates a 95% confidence interval around the fit. The associations were generally linear, with *P*-values for nonlinearity of .08 for CI, .63 for ABSI, .31 for RFM, and .25 for WHtR. Panels: (A) CI *z*-scores, (B) ABSI *z*-scores, (C) RFM *z*-scores, (D) WHtR *z*-scores. ABSI = A Body Shape Index, CI = conicity index, FI = fecal incontinence, PIR = poverty-to-income ratio, RFM = relative fat mass, WHtR = waist-to-height ratio.

The dose–response relationships assessed using RCS are shown in Figure 2. The associations between the *z*-scores of CI, ABSI, RFM, and WHtR and the log odds of FI were generally linear, with *P*-values for nonlinearity of .08, .63, .31, and .25, respectively.

### 3.4. Subgroup analysis

**Figure 3.**
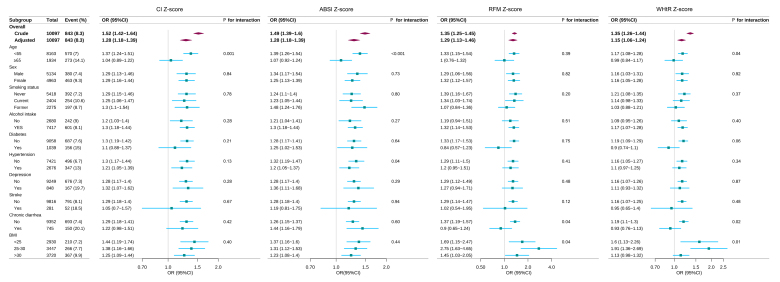
Subgroup analyses and interaction tests for associations between anthropometric indices and fecal incontinence (National Health and Nutrition Examination Survey, 2005–2010 cycles). The analyses were performed using models that controlled for the following covariates: age, sex, race, education level, marital status, PIR, physical activity, smoking status, alcohol intake, total fat intake, diabetes, hypertension, depression, chronic diarrhea, constipation, and stroke. When the results were reported according to the strata, the stratification variable itself was not included among the adjusted covariates. Positive associations were observed in most subgroups, with significant effect modifications noted for age (CI and ABSI), BMI (RFM and WHtR), and chronic diarrhea status (WHtR). 95% CI = 95% confidence interval, ABSI = A Body Shape Index, CI = conicity index, OR = odds ratio, PIR = poverty-to-income ratio, RFM = relative fat mass, WHtR = waist-to-height ratio.

The results of the subgroup analyses are shown in Figure 3. An interaction by age was observed for the association of CI and ABSI with FI (*P* for interaction < .001), with stronger associations among participants younger than 65 years of age (CI: OR, 1.37; 95% CI: 1.24–1.51; ABSI: OR, 1.39; 95% CI: 1.26–1.54), but weaker among participants aged ≥65 years (CI: OR, 1.04; 95% CI: 0.89–1.22; ABSI: OR, 1.07; 95% CI: 0.92–1.24). The association between RFM and FI also varied by BMI category (P for interaction = 0.04), with the strongest association seen in the overweight subgroup (BMI 25–30 kg/m^2^: OR, 2.75; 95% CI: 1.63–4.65). For the WHtR, interactions were found with age (P for interaction = 0.04), BMI (P for interaction = 0.01), and chronic diarrhea status (P for interaction = 0.02). However, in the majority of subgroups, these indices showed a consistent positive correlation with FI.

### 3.5. ROC analysis

**Figure 4.**
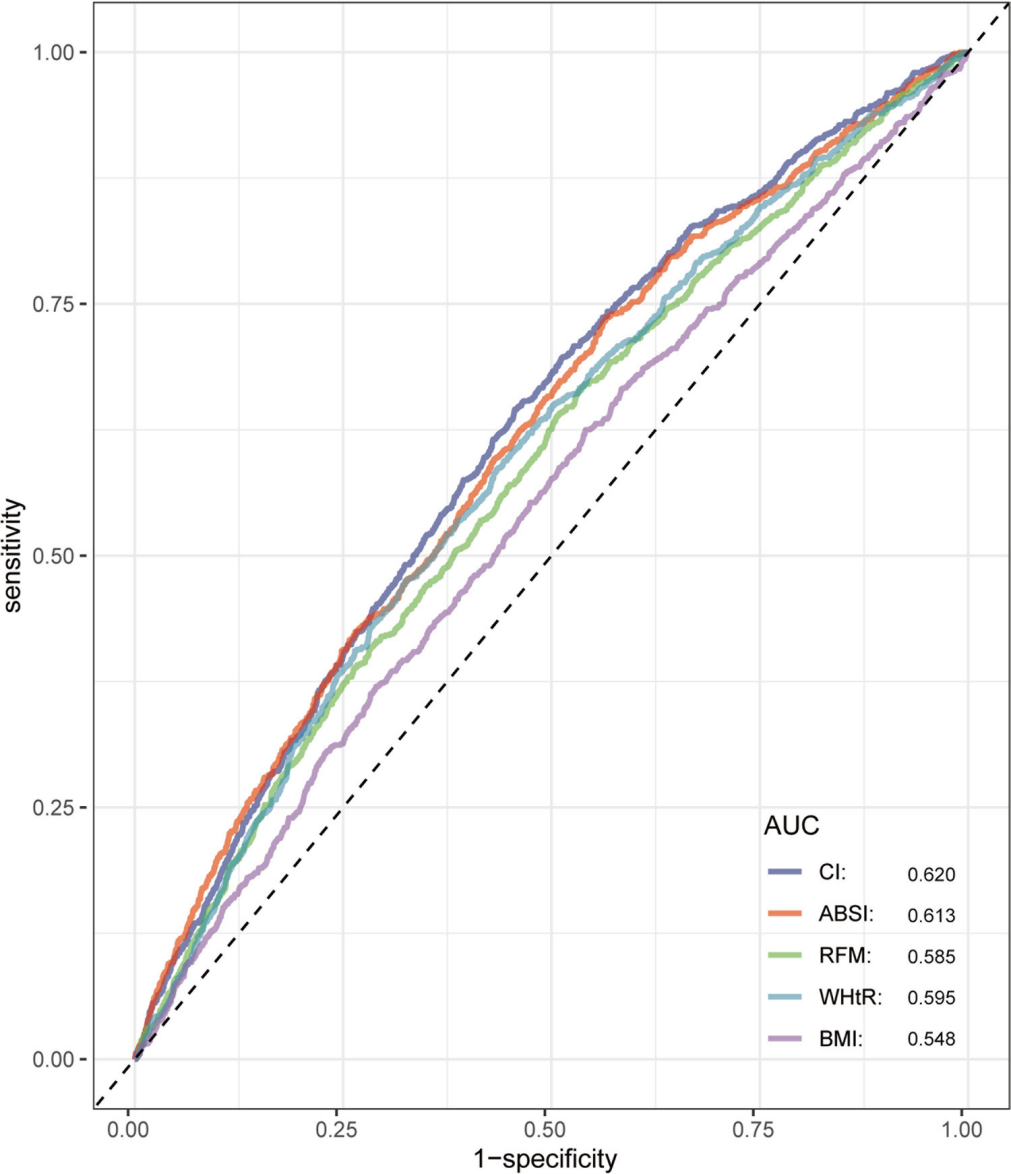
ROC curve for fecal incontinence (National Health and Nutrition Examination Survey, 2005–2010 cycles). ROC curves and AUC values for anthropometric indices (CI, ABSI, RFM, WHtR, and BMI) were used to distinguish fecal incontinence status. The discriminatory ability was modest for all indices, with the highest AUC values observed for CI (0.620) and ABSI (0.613), followed by WHtR (0.595), RFM (0.585), and BMI (0.548). ABSI = A Body Shape Index, AUC = area under the curve, BMI = body mass index, CI = conicity index, RFM = relative fat mass, WHtR = waist-to-height ratio, ROC = receiver operating characteristic.

The AUC values for the anthropometric indices were as follows: CI = 0.620 (95% CI: 0.60–0.64), ABSI = 0.613 (95% CI: 0.59–0.63), WHtR = 0.595 (95% CI: 0.58–0.62), RFM = 0.585 (95% CI: 0.57–0.61), and BMI = 0.548 (95% CI: 0.53–0.57; Fig. 4 and Table S3, Supplemental Digital Content).

## 4. Discussion

We comprehensively evaluated and compared the associations between 5 anthropometric indices (CI, ABSI, RFM, WHtR, and BMI) and FI. This cross-sectional study used data from the 2005 to 2010 cycles of NHANES and included 10,097 participants. We found that CI, ABSI, RFM, and WHtR were linearly and positively associated with FI in the fully adjusted model; however, BMI did not show this association. While CI and ABSI showed relatively stronger associations with FI among the 5 indices, the discriminatory ability of all indices was limited. These associations were not completely consistent in different subgroups, and the association between CI and ABSI were stronger in adults under 65 years of age. The associations of RFM and WHtR differed across BMI categories, but the positive association trend remained in the majority of subgroups.

Our study showed that BMI was not significantly associated with FI, whereas WHtR was positively and significantly associated with FI. In our study, whether BMI was used as a continuous variable or stratified by quartile, it did not show a significant association with FI in the fully adjusted model (OR: 1.05, 95% CI: 0.98–1.13, *P* = .19). This finding is consistent with those of several previous studies. For example, a prospective cohort analysis of the Nurses’ Health Study showed no independent association between BMI and FI after adjusting for physical activity, diabetes, and other factors.^[12]^ Similarly, a cross-sectional analysis of NHANES 2005 to 2006 data by Whitehead et al reported no significant association between BMI and FI after multivariate adjustment.^[14]^ In contrast, the current study extends the analysis by using data from the NHANES 2005 to 2010 period, which had a larger sample size, further enhancing the robustness of the findings. We also explored the relationship between indices of central obesity, such as WHtR, CI, ABSI, and RFM, with FI. Our study found that WHtR was independently and positively correlated with FI (OR: 1.15, 95% CI: 1.06–1.24, *P* < .001), and it showed stronger associations and slightly higher AUC values than BMI, which was consistent with the results of Hiramoto et al.^[15]^ The inconsistent association among BMI, WHtR, and FI may be because BMI mainly measures the degree of overall obesity, and it is difficult to effectively distinguish fat distribution, whereas WHtR reflects the accumulation of abdominal fat. Therefore, abdominal obesity rather than overall obesity may be more closely associated with FI.

We found a positive linear association between the CI, ABSI, RFM, and FI. These indices were designed to overcome the limitations of BMI by considering body proportions to quantify central obesity.^[16]^ ABSI is based on the allometric relationship between WC, height, and weight, which effectively reduces the collinearity between WC and BMI, suggesting the risk of abdominal fat accumulation.^[14]^ RFM is estimated based on the ratio of height to WC, which is more accurate in reflecting the percentage of body fat and has good consistency across different sexes and races. Based on the geometric model of a cone, the CI quantifies the degree of concentric accumulation of fat by comparing the WC with the theoretical circumference calculated from height and weight and is a more sensitive representation of central obesity. The consistency of these associations was further reinforced by clear dose–response gradients in our sensitivity analyses, with participants in the highest quartile of CI, ABSI, and RFM having significantly higher odds of FI than those in the lowest quartile (CI: OR = 1.86; ABSI: OR = 1.90; RFM: OR = 1.88). This pattern was also confirmed in continuous analyses (Model 3: CI OR: 1.28, 95%CI: 1.18–1.39, *P* < .001; ABSI OR: 1.28, 95% CI: 1.18–1.39, *P* < .001), and RCS analysis further confirmed a linear dose–response relationship between FI and these indices. Although studies directly addressing the association between these indices and FI are limited, our findings are mechanistically consistent with those linking visceral adiposity to pelvic floor dysfunction. The performance of these novel indices in other pelvic floor disorders provides supporting evidence. For example, a cross-sectional study found that for each 1-SD increase in ABSI and CI, the risk of erectile dysfunction increased by 49% and 42%, respectively.^[33]^ A cohort study in women with urinary incontinence showed that CI was significantly associated with the incidence and clinical severity of urinary incontinence.^[26]^ In addition, Liu reported that ABSI and RFM were more strongly associated with overactive bladder than was BMI.^[25]^ These pelvic floor disorders may share underlying pathophysiological pathways with FI. The putative mechanisms may involve increased intra-abdominal pressure, leading to mechanical traction of the pelvic floor structures, as well as systemic disorders such as chronic low-grade inflammation and impaired neurovascular integrity. These novel indices effectively reflect central fat deposition and are significantly related to the odds of FI.

Our subgroup analyses confirmed positive associations across most strata, supporting the generalizability of our results. However, we identified important effects of modification. The associations of CI and ABSI with FI were more significant in younger people (<65 years), indicating that the association between obesity and FI is more prominent in younger people. This phenomenon may be related to the fact that the older population is more affected by age-related comorbidities and functional decline. The association between RFM and FI was stronger in the overweight population than in other BMI groups. This suggests that the association between fat distribution characteristics and FI differs across BMI groups. These interactions suggest that the relationship between specific fat distribution patterns and FI may vary across population groups and warrant further investigation.

ROC analysis showed that CI and ABSI had slightly higher AUC values for FI among the 5 anthropometric indices. However, all AUC values were modest (<0.70), indicating that no single index alone can accurately predict FI. The relative ranking of AUC suggested that CI and ABSI had marginally better discriminatory ability than WHtR, RFM, and BMI, but their limited discriminatory ability suggests they are more appropriate as components of a composite risk assessment tool than as standalone screening tests.

The association between obesity and FI involves several complex biological mechanisms. Abdominal fat accumulation can increase intra-abdominal pressure and compress pelvic floor structures, leading to the relaxation of the rectal supporting ligament and decreased anal sphincter function, thereby impairing bowel control.^[34–36]^ Obesity increases the rectal sensory threshold, delays defecation perception, and impairs bowel control.^[37]^ In addition, visceral fat, as an endocrine organ, secretes proinflammatory factors (such as tumor necrosis factor-α and interleukin-6) and dysregulated adipokines, causing chronic inflammation and metabolic disorders and damaging the neuromuscular structure of the pelvic floor.^[36,38,39]^ The intestinal function changes caused by obesity, such as diarrhea, accelerated intestinal transport, and intestinal flora imbalance, may reduce the ability to control defecation.^[40–42]^ In addition, anal sphincter injury caused by anal surgery and obstetric trauma is also an important factor leading to FI.^[43,44]^ Although the exact mechanism by which obesity leads to FI has not yet been fully elucidated, some studies have shown that weight loss can alleviate FI.^[45]^ Therefore, weight loss measures targeting abdominal fat may have greater significance in improving FI symptoms.

Data from NHANES (2005–2010) were used in this study. Thus, the adequate sample size supported the robustness of our findings. Using multivariate logistic regression models, RCS analysis, and ROC curve evaluation, this study confirmed that the novel anthropometric indices were significantly correlated with FI. These measures are better at reflecting patterns of body fat distribution, especially visceral fat, than the traditional BMI. There is a lack of previous studies directly exploring the correlation between these indices and FI. This study highlights the association between central adiposity and FI and suggests that further research is needed to explore the potential role of these indices in risk assessment.

This study had some limitations. The cross-sectional design limits causal inference and needs to be validated in prospective studies. Our analyses did not incorporate the NHANES complex sampling weights, and the results are representative of the analyzed sample but not the US population. Selection bias may have been present because participants who were excluded due to missing data had higher levels of central obesity and a greater burden of coexisting conditions. This may have resulted in underestimating the observed strength of the associations. Self-reported covariates may introduce recall bias, and the US-based sample limits the generalizability of the results to other ethnic groups. Despite adjusting for multiple confounders, residual confounding from unmeasured factors such as obstetric history, anorectal surgery, and medication use cannot be completely ruled out. The modest AUC values (<0.70) indicate these indices have limited utility as standalone screening tools. Future prospective studies are needed to validate these associations, explore the underlying pathophysiological pathways, and assess the clinical utility of these novel anthropometric indices.

In conclusion, this cross-sectional study showed that the indices of central adiposity (CI, ABSI, RFM, and WHtR), but not BMI, were significantly and positively correlated with FI. Among these 5 indices, although CI and ABSI showed relatively stronger associations with FI compared to the others, their discriminatory ability was limited. Compared with general obesity, central fat distribution is more closely related to FI. The underlying mechanism may involve pathophysiological pathways such as elevated intra-abdominal pressure and chronic inflammation. Future prospective studies are needed to validate these associations, explore the underlying mechanisms, and assess their clinical utility.

## Acknowledgments

The authors thank the NHANES research team, staff, and participants for their invaluable contributions. We also extend our thanks to the Free Statistics team (Beijing, China) for their technical assistance and provision of data analysis and visualization tools.

## Author contributions

**Methodology:** Zhijun Ye, Guorong Huang, Erdong Chen.

**Formal analysis:** Zhijun Ye, Lijuan Huang, Xue Zhang.

**Project administration:** Zhijun Ye, Limin Tao.

**Software:** Zhijun Ye, Xue Zhang.

**Supervision:** Zhijun Ye, Limin Tao.

**Resources:** Guorong Huang, Xiaoyun Lan, Yanghua Hong.

**Writing – original draft:** Zhijun Ye, Guorong Huang, Xiaoyun Lan, Lijuan Huang.

**Writing – review & editing:** Erdong Chen, Yanghua Hong, Limin Tao.






