# Supplementary material for: Associations between five anthropometric indices and fecal incontinence: A cross-sectional study based on the 2005 to 2010 NHANES data
Source: Medicine (Baltimore). 2026 Jun 5;105(23):e49139. doi: 10.1097/MD.0000000000049139 (PMC13246086; doi:10.1097/MD.0000000000049139)
Supplement: Supplementary file 1 [file medi-105-e49139-s001.docx]

**Table S1. Baseline characteristics of anthropometric indicators after z-score transformation,stratified by fecal incontinence status (National Health and Nutrition Examination Survey, 2005–2010 cycles) .**

| **Characteristics** | **Total(n = 10097)** | **Without FI(n = 9254)** | **With FI(n = 843)** | ***P-value*** |
| --- | --- | --- | --- | --- |
| CI z-score, Mean (SD) | 0.00 ± 1.00 | -0.03 ± 1.00 | 0.38 ± 0.96 | < .001 |
| ABSI z-score, Mean (SD) | 0.00 ± 1.00 | -0.03 ± 0.99 | 0.37 ± 0.99 | < .001 |
| RFM z-score, Mean (SD) | 0.00 ± 1.00 | -0.02 ± 1.00 | 0.27 ± 0.99 | < .001 |
| WHtR z-score, Mean (SD) | 0.00 ± 1.00 | -0.03 ± 1.00 | 0.29 ± 1.01 | < .001 |
| BMI z-score,Mean (SD) | 0.00 ± 1.00 | -0.01 ± 1.00 | 0.14 ± 1.04 | < .001 |
| WC z-score, Mean (SD) | 0.00 ± 1.00 | -0.02 ± 1.00 | 0.24 ± 1.01 | < .001 |

Continuous variables are presented as mean ± standard deviation (SD).

FI = fecal incontinence,CI = conicity index, ABSI = a body shape index,RFM = relative fat mass,WHtR = waist-to-height ratio, BMI = body mass index,WC = waist circumference.
