# Supplementary material for: Associations between five anthropometric indices and fecal incontinence: A cross-sectional study based on the 2005 to 2010 NHANES data
Source: Medicine (Baltimore). 2026 Jun 5;105(23):e49139. doi: 10.1097/MD.0000000000049139 (PMC13246086; doi:10.1097/MD.0000000000049139)
Supplement: Supplementary file 2 [file medi-105-e49139-s002.docx]

**Table S2. Baseline characteristics of included and excluded participants (National Health and Nutrition Examination Survey, 2005–2010 cycles) .**

| **Characteristics** | **Total**  **(n = 17132)** | **Excluded**  **(n = 7035)** | **Included**  **(n = 10097)** | ***P-value*** |
| --- | --- | --- | --- | --- |
| Age(year), n (%) |  |  |  | < .001 |
| <65 | 12864 (75.09) | 4701 (66.82) | 8163 (80.85) |  |
| ≥65 | 4268 (24.91) | 2334 (33.18) | 1934 (19.15) |  |
| Sex, n (%) |  |  |  | < .001 |
| Male | 8303 (48.46) | 3169 (45.05) | 5134 (50.85) |  |
| Female | 8829 (51.54) | 3866 (54.05) | 4963 (49.15) |  |
| Race, n (%) |  |  |  | .02 |
| Non-Hispanic white | 8232 (48.05) | 3300 (46.91) | 4932 (48.85) |  |
| Non-Hispanic black | 3472 (20.27) | 1428 (20.3) | 2044 (20.24) |  |
| Mexican American | 3176 (18.54) | 1376 (19.56) | 1800 (17.83) |  |
| Others | 2252 (13.14) | 931 (13.23) | 1321 (13.08) |  |
| Education level(year), n(%) |  |  |  | < .001 |
| <9 | 2210 (12.92) | 1186 (16.93) | 1024 (10.14) |  |
| 9-12 | 6890 (40.29) | 2942 (42) | 3948 (39.1) |  |
| >12 | 8001 (46.79) | 2876 (41.06) | 5125 (50.76) |  |
| Marital status,n (%) |  |  |  | .001 |
| Married or living With a partner | 10281 (60.06) | 4116 (58.63) | 6165 (61.06) |  |
| Living alone | 6836 (39.94) | 2904 (41.37) | 3932 (38.94) |  |
| Poverty income ratio, n (%) |  |  |  | < .001 |
| Low | 4775 (30.43) | 1835 (32.79) | 2940 (29.12) |  |
| Medium | 6096 (38.85) | 2252 (40.24) | 3844 (38.07) |  |
| High | 4822 (30.73) | 1509 (26.97) | 3313 (32.81) |  |
| Physical activity, n (%) |  |  |  | < .001 |
| Sedentary | 3634 (23.98) | 1535 (30.37) | 2099 (20.79) |  |
| Insufficient | 2535 (16.73) | 843 (16.68) | 1692 (16.76) |  |
| Moderate | 1813 (11.97) | 586 (11.59) | 1227 (12.15) |  |
| High | 7170 (47.32) | 2091 (41.36) | 5079 (50.3) |  |
| Smoking status, n (%) |  |  |  | < .001 |
| Never | 9104 (53.18) | 3686 (52.49) | 5418 (53.66) |  |
| Current | 4261 (24.89) | 1857 (26.45) | 2404 (23.81) |  |
| Former | 3754 (21.93) | 1479 (21.06) | 2275 (22.53) |  |
| Alcohol intake, n (%) |  |  |  | < .001 |
| No | 4335 (29.08) | 1655 (34.42) | 2680 (26.54) |  |
| Yes | 10570 (70.92) | 3153 (65.58) | 7417 (73.46) |  |
| Total fat intake (g/d), Median (IQR) | 69.22 (45.81, 100.11) | 65.70 (43.96, 95.81) | 71.46 (47.01, 102.78) | < .001 |
| **Chronic diseases** |  | | | |
| Diabetes, n (%) |  |  |  | < .001 |
| No | 15125 (88.37) | 6067 (86.44) | 9058 (89.71) |  |
| Yes | 1991 (11.63) | 952 (13.56) | 1039 (10.29) |  |
| Hypertension, n (%) |  |  |  | < .001 |
| No | 12231 (71.65) | 4810 (68.97) | 7421 (73.5) |  |
| Yes | 4840 (28.35) | 2164 (31.03) | 2676 (26.5) |  |
| Depression, n (%) |  |  |  | < .001 |
| No | 13470 (90.86) | 4221 (89.28) | 9249 (91.6) |  |
| Yes | 1355 ( 9.14) | 507 (10.72) | 848 (8.4) |  |
| Chronic diarrhea, n (%) |  |  |  | .004 |
| No | 13478 (92.20) | 4126 (91.24) | 9352 (92.62) |  |
| Yes | 1141 ( 7.80) | 396 (8.76) | 745 (7.38) |  |
| Constipation, n (%) |  |  |  | .003 |
| No | 13488 (92.26) | 4128 (91.29) | 9360 (92.7) |  |
| Yes | 1131 ( 7.74) | 394 (8.71) | 737 (7.3) |  |
| Stroke, n (%) |  |  |  | < .001 |
| No | 16419 (96.03) | 6603 (94.33) | 9816 (97.22) |  |
| Yes | 678 ( 3.97) | 397 (5.67) | 281 (2.78) |  |
| **Anthropometric indices** |  | | | |
| Weight.kg, Mean(SD) | 81.38 ± 21.22 | 80.08 ± 21.50 | 82.18 ± 21.01 | < .001 |
| Height.cm, Mean (SD) | 167.31 ± 10.20 | 165.90 ± 10.12 | 168.19 ± 10.15 | < .001 |
| WC.cm, Mean (SD) | 98.83 ± 15.91 | 99.16 ± 15.55 | 98.64 ± 16.10 | .05 |
| CI, Mean (SD) | 1.31 ± 0.09 | 1.32 ± 0.09 | 1.30 ± 0.09 | < .001 |
| ABSI, Mean (SD) | 0.08 ± 0.01 | 0.08 ± 0.01 | 0.08 ± 0.01 | < .001 |
| RFM, Mean (SD) | 35.50 ± 8.54 | 36.47 ± 8.42 | 34.97 ± 8.57 | < .001 |
| WHtR, Mean (SD) | 0.59 ± 0.10 | 0.60 ± 0.09 | 0.59 ± 0.10 | < .001 |
| BMI.kg/m^2^, Mean (SD) | 28.98 ± 6.76 | 29.00 ± 7.00 | 28.97 ± 6.60 | .73 |

Data are presented as number (percentage) for categorical variables and as mean ± standard deviation (SD) or median (interquartile range, IQR) for continuous variables.

FI = fecal incontinence,WC = waist circumference,CI = conicity index,ABSI = a body shape index,RFM = relative fat mass,WHtR = waist-to-height ratio,BMI = body mass index.
