# Supplementary material for: Associations between five anthropometric indices and fecal incontinence: A cross-sectional study based on the 2005 to 2010 NHANES data
Source: Medicine (Baltimore). 2026 Jun 5;105(23):e49139. doi: 10.1097/MD.0000000000049139 (PMC13246086; doi:10.1097/MD.0000000000049139)
Supplement: Supplementary file 3 [file medi-105-e49139-s003.docx]

**Table S3. AUC comparison of anthropometric indicators for fecal incontinence (National Health and Nutrition Examination Survey, 2005–2010 cycles) .**

| **Test** | **AUC^a^** | **95%CI^b^ low** | **95%CI up** | **Best threshold** | **Specificity** | **Sensitivity** | ***P*^c^ for different in AUC** |
| --- | --- | --- | --- | --- | --- | --- | --- |
| BMI | 0.548 | 0.59 | 0.57 | 27.29 | 0.46 | 0.63 | Reference |
| CI | 0.620 | 0.60 | 0.64 | 1.31 | 0.54 | 0.66 | < .001 |
| ABSI | 0.613 | 0.59 | 0.63 | 0.08 | 0.44 | 0.74 | < .001 |
| RFM | 0.585 | 0.57 | 0.61 | 33.87 | 0.49 | 0.64 | < .001 |
| WHtR | 0.595 | 0.58 | 0.62 | 0.59 | 0.57 | 0.59 | < .001 |

**^a^**AUC, area under the receiver operating characteristic (ROC) curve. ^b^95%CI, 95% confidence interval.^c^*P*, *P*-value.

FI = fecal incontinence,BMI = body mass index,CI = conicity index,ABSI = a body shape index,RFM = relative fat mass,WHtR = waist-to-height ratio.
